# Psychological, clinical and pathological effects of relaxation training and guided imagery during primary chemotherapy

**DOI:** 10.1038/sj.bjc.6690349

**Published:** 1999-04

**Authors:** L G Walker, M B Walker, K Ogston, S D Heys, A K Ah-See, I D Miller, A W Hutcheon, T K Sarkar, O Eremin

**Affiliations:** Behavioural Oncology Unit, Department of Surgery, Surgical Nutrition and Metabolism Unit, Department of Pathology and Department of Medicine & Therapeutics, University of Aberdeen, Medical School, Foresterhill, Aberdeen AB25 2ZD, UK

**Keywords:** chemotherapy, breast cancer, quality of life, randomized trial

## Abstract

The diagnosis and treatment of breast cancer are stressful, and stress may be associated with a poorer response to chemotherapy. There is a need, therefore, to develop and evaluate interventions that might enhance quality of life and, possibly, improve treatment response. The effects of relaxation combined with guided imagery (visualizing host defences destroying tumour cells) on quality of life and response to primary chemotherapy, to date, have not been adequately evaluated. Ninety-six women with newly diagnosed large or locally advanced breast cancer (T_2_ > 4 cm, T_3_, T_4_, or T_x_N_2_ and M_0_) took part in a prospective, randomized controlled trial. Patients were randomized following diagnosis to a control condition (standard care) or to the experimental condition (standard care plus relaxation training and imagery). Psychometric tests to evaluate mood and quality of life were carried out before each of the six cycles of chemotherapy and 3 weeks after cycle 6: tests of personality and coping strategy were carried out prior to cycles one and six. Clinical response to chemotherapy was evaluated after six cycles of chemotherapy using standard UICC criteria and pathological response was assessed from the tissue removed at surgery. As hypothesized, patients in the experimental group were more relaxed and easy going during the study (Mood Rating Scale). Quality of life was better in the experimental group (Global Self-assessment and Rotterdam Symptom Checklist). The intervention also reduced emotional suppression (Courtauld Emotional Control Scale). The incidence of clinically significant mood disturbance was very low and the incidence in the two groups was similar. Finally, although the groups did not differ for clinical or pathological response to chemotherapy, imagery ratings were correlated with clinical response. These simple, inexpensive and beneficial interventions should be offered to patients wishing to improve quality of life during primary chemotherapy. © 1999 Cancer Research Campaign


*British Journal of Cancer* (1999) **80**(1/2), 262–268
© 1999 Cancer Research Campaign
Article no. bjoc.1998.0349

### Psychological, clinical and pathological effects of relaxation training and guided imagery during primary chemotherapy

**LG Walker[1], MB Walker[1], K Ogston[2], SD Heys[3], AK Ah-See[2], ID Miller[4], AW Hutcheon[5], TK Sarkar[2] and O Eremin[2]**

[1]Behavioural Oncology Unit, [2]Department of Surgery, [3]Surgical Nutrition and Metabolism Unit, [4]Department of Pathology and [5]Department of Medicine & Therapeutics, University of Aberdeen, Medical School, Foresterhill, Aberdeen AB25 2ZD, UK

**Summary** The diagnosis and treatment of breast cancer are stressful, and stress may be associated with a poorer response to chemotherapy. There is a need, therefore, to develop and evaluate interventions that might enhance quality of life and, possibly, improve treatment response. The effects of relaxation combined with guided imagery (visualizing host defences destroying tumour cells) on quality of life and response to primary chemotherapy, to date, have not been adequately evaluated. Ninety-six women with newly diagnosed large or locally advanced breast cancer ($T_2$ > 4 cm, $T_3$, $T_4$, or $T_xN_2$ and $M_0$) took part in a prospective, randomized controlled trial. Patients were randomized following diagnosis to a control condition (standard care) or to the experimental condition (standard care plus relaxation training and imagery). Psychometric tests to evaluate mood and quality of life were carried out before each of the six cycles of chemotherapy and 3 weeks after cycle 6: tests of personality and coping strategy were carried out prior to cycles one and six. Clinical response to chemotherapy was evaluated after six cycles of chemotherapy using standard UICC criteria and pathological response was assessed from the tissue removed at surgery. As hypothesized, patients in the experimental group were more relaxed and easy going during the study (Mood Rating Scale). Quality of life was better in the experimental group (Global Self-assessment and Rotterdam Symptom Checklist). The intervention also reduced emotional suppression (Courtauld Emotional Control Scale). The incidence of clinically significant mood disturbance was very low and the incidence in the two groups was similar. Finally, although the groups did not differ for clinical or pathological response to chemotherapy, imagery ratings were correlated with clinical response. These simple, inexpensive and beneficial interventions should be offered to patients wishing to improve quality of life during primary chemotherapy.

**Keywords:** chemotherapy; breast cancer; quality of life; randomized trial

It is well-established that the diagnosis and treatment of breast cancer are stressful experiences that may be associated with high levels of psychiatric and psychosocial morbidity during the first year following diagnosis (Maguire et al, 1978). Although adaptation and quality of life can be enhanced by giving patients information appropriately and by involving them in decision-making to the extent they wish (Fallowfield, 1997), there is clearly a need for simple, easily administered interventions to help women to cope with the diagnosis and treatment of breast cancer (Walker and Eremin, 1996).

There is substantial evidence that quality of life is an independent prognostic factor for survival in various types of cancer (Weeks, 1992; Ratcliffe et al, 1995; Coates et al, 1997). Moreover, various aspects of quality of life may independently predict response to chemotherapy. For example, in patients with advanced breast cancer who underwent chemotherapy, Fraser et al (1993) found that quality of life at trial entry predicted response to chemotherapy and, subsequently, survival. However, it is not known if psychological interventions designed to improve quality of life can enhance response to adjuvant or neoadjuvant chemotherapy for breast cancer.

*Received 30 June 1998*
*Revised 12 October 1998*
*Accepted 5 November 1998*

*Correspondence to:* LG Walker

The most convincing evidence for the importance of psychosocial factors in modulating tumour biology comes from psychosocial intervention studies. Spiegel et al (1989) found a doubling in mean (although not median) survival in women with metastatic breast cancer randomized to a group intervention. Beneficial effects of psychological interventions on survival have also been demonstrated in patients with malignant melanoma (Fawzy et al, 1990) and haematological malignancies (Richardson et al, 1990; Ratcliffe et al, 1995; Walker, 1998, Walker et al, 1998).

There is a need, therefore, to develop and evaluate interventions that minimize the psychosocial impact of the diagnosis and treatment of cancer and that may enhance clinical and pathological responses to chemotherapy (Dreher, 1997).

Relaxation therapy is used extensively with patients who have cancer (Walker, 1992; Downer et al, 1994). In addition to studies of the effects of relaxation therapy on treatment-induced nausea, vomiting and related side-effects (Burish et al, 1988), two randomized, controlled studies of the psychological effects of relaxation have been reported in patients with breast cancer. Bridge et al (1988) evaluated the effects of relaxation with, or without, ‘peaceful’ imagery in 154 women receiving radiotherapy as outpatients. Compared with the control group, total mood disturbance was significantly less in the two intervention groups. The combination of relaxation and peaceful imagery was significantly better than relaxation on its own. The second randomized study was very small (Walker, 1992; Gruber et al, 1993). Thirteen patients with stage 1 breast cancer were randomized to a delayed treatment

**Table 1** Pre- and post-chemotherapy scores for the control group (C) and the experimental group (E)

| | | | Pre-chemotherapy | | Post-chemotherapy | | Repeated measures analysis of variance/covariance | | | | | |
|---|---|---|---|---|---|---|---|---|---|---|---|---|
| | | | | | | | Between groups | | Time | | Group and time interaction | |
| | | | Mean | s.d. | Mean | s.d. | F | *P* | F | *P* | F | *P* |
| CECS | total | C | 51.50 | (9.69) | 52.13 | (9.63) | 2.02 | 0.16 | 2.28 | 0.14 | 5.24 | 0.02 |
| | | E | 50.63 | (10.52) | 47.58 | (10.64) | | | | | | |
| | Anger | C | 16.33 | (3.63) | 15.88 | (3.49) | 0.42 | 0.52 | 2.53 | 0.12 | 0.10 | 0.75 |
| | | E | 15.98 | (4.46) | 15.29 | (4.23) | | | | | | |
| | Unhappiness | C | 17.21 | (4.34) | 18.00 | (3.87) | 3.09 | 0.08 | 0.14 | 0.71 | 6.33 | 0.01 |
| | | E | 16.83 | (3.92) | 15.77 | (4.07) | | | | | | |
| | Anxiety | C | 18.04 | (4.00) | 18.04 | (3.89) | 1.54 | 0.22 | 3.67 | 0.06 | 3.67 | 0.06 |
| | | E | 17.81 | (3.98) | 16.44 | (4.23) | | | | | | |
| EPQ-L | | C | 11.31 | (3.91) | 11.67 | (4.37) | 0.54 | 0.46 | 0.20 | 0.65 | 2.99 | 0.09 |
| | | E | 11.17 | (4.48) | 10.56 | (4.69) | | | | | | |
| GQOL | | C | 3.23 | (0.66) | 2.90 | (0.78) | 2.81 | 0.10 | 2.97 | 0.09 | 4.91 | 0.03 |
| | | E | 3.25 | (0.70) | 3.29 | (0.80) | | | | | | |
| MRS | Relaxation | C | 71.56 | (45.14) | 70.19 | (41.30) | 3.61 | 0.06 | 3.74 | 0.004 | 3.06 | 0.01 |
| | | E | 57.98 | (42.98) | 73.29 | (44.77) | | | | | | |
| | Easy-going | C | 88.15 | (44.06) | 85.33 | (44.07) | 5.31 | 0.02 | 1.47 | 0.21 | 0.80 | 0.55 |
| | | E | 73.29 | (38.49) | 100.00 | (36.62) | | | | | | |
| | Happiness | C | 78.56 | (36.47) | 92.15 | (34.01) | 2.62 | 0.11 | 0.49 | 0.78 | 0.82 | 0.54 |
| | | E | 79.63 | (38.90) | 94.15 | (31.22) | | | | | | |
| | Energy | C | 70.31 | (34.56) | 69.81 | (34.81) | 0.49 | 0.49 | 5.88 | 0.0005 | 0.50 | 0.78 |
| | | E | 67.73 | (40.30) | 77.18 | (41.43) | | | | | | |
| | Confusion | C | 117.56 | (39.00) | 120.90 | (35.68) | 0.79 | 0.38 | 1.34 | 0.26 | 0.96 | 0.45 |
| | | E | 107.44 | (41.26) | 112.89 | (33.67) | | | | | | |
| | Confidence | C | 88.48 | (44.33) | 89.98 | (44.17) | 0.05 | 0.83 | 2.41 | 0.04 | 0.32 | 0.90 |
| | | E | 87.50 | (42.63) | 91.21 | (39.46) | | | | | | |
| | Total | C | 514.63 | (171.15) | 528.35 | (154.27) | 4.82 | 0.03 | 1.14 | 0.34 | 0.89 | 0.49 |
| | | E | 473.56 | (151.87) | 548.79 | (149.59) | | | | | | |
| RSCL | Psychological | C | 12.54 | (2.97) | 11.50 | (2.60) | 3.70 | 0.06 | 1.48 | 0.21 | 0.40 | 0.85 |
| | | E | 13.33 | (4.10) | 11.42 | (3.03) | | | | | | |
| | Physical | C | 27.85 | (5.56) | 30.54 | (7.00) | 0.01 | 0.93 | 10.84 | 0.0005 | 0.36 | 0.87 |
| | | E | 28.40 | (5.00) | 31.10 | (5.74) | | | | | | |
| HADS Anxiety | | C | 2.73 | (0.77) | 2.32 | (0.72) | 0.23 | 0.64 | 2.68 | 0.03 | 1.48 | 0.20 |
| (1 + square root) | | E | 2.71 | (0.7) | 2.25 | (0.72) | | | | | | |
| HADS Depression | | C | 1.79 | (0.65) | 1.90 | (0.73) | 0.87 | 0.35 | 5.70 | 0.0005 | 0.31 | 0.90 |
| (1 + square root) | | E | 1.76 | 0.61) | 1.86 | (0.67) | | | | | | |

control or to group relaxation training, relaxing imagery and electromyographic biofeedback. Although immunological and biochemical changes were observed, a between-group analysis did not find any significant changes in quality of life or other psychological measures. Studies with mixed groups of cancer patients have reported beneficial effects on mood (Burish et al, 1988; Bindemann et al, 1991; Holland et al, 1991; Baider et al, 1994).

It may be that relaxation therapy enhances ability to cope with stress. No randomized studies have directly assessed this, although Burish et al (1988) followed up 50 cancer patients who had been taught progressive muscular relaxation to reduce chemotherapy side-effects. They found that 97% of the respondents would recommend the technique as an effective coping strategy and they reported using the technique for a variety of specific problems, including ‘nervousness’, insomnia and pain.

Like relaxation, visualization is a commonly used form of complementary medicine: Downer et al (1994) found that visualization was the third most common complementary technique used by an unselected series of 600 patients in the UK. Gruber et al (1988) studied ten adults with metastatic cancer. Following two baseline sessions, relaxation and guided imagery training (visualizing host defences destroying malignant cells) were carried out. Audiotapes were also used, as were group sessions and electromyographic feedback. Changes in lymphocyte mitogen responsiveness, interleukin-2 secretion, natural killer (NK) cell activity and production of immunoglobulins were documented. However, the psychobiological effects of guided imagery alone, or in combination with relaxation therapy, have not been evaluated in a randomized study (Simonton et al, 1980).

### Hypotheses

Compared with controls receiving the same amount of staff support, patients randomized to relaxation and guided imagery will have better quality of life and improved coping skills. Their clinical and pathological responses to chemotherapy will be superior and the frequency of relaxation practice will be positively correlated with response to chemotherapy.

### METHODS

### Inclusion and exclusion criteria and investigations

The inclusion criteria were: newly diagnosed large or locally advanced breast cancer ($T_2$ > 4 cm, $T_3$, $T_4$ or $N_2$ node status and $M_0$

*© Cancer Research Campaign 1999* 

**Table 2** Patient characteristics (by group) at trial entry

| | Control | Experimental | *P*-value |
|---|---|---|---|
| Mean age (s.d.) | 50.1(11.3) | 49.3(10.8) | 0.71 |
| Tumour stage (numbers) | | | |
| $T_2$ | 14 | 15 | 0.47 |
| $T_3$ | 24 | 28 | |
| $T_4$ | 9 | 5 | |
| $T_x$ | 1 | | |
| Clinical nodal status | | | |
| $N_0$ | 29 | 29 | 0.35 |
| $N_1$ | 15 | 18 | |
| $N_2$ | 4 | 1 | |
| Menopausal status | | | |
| Premenopausal | 27 | 24 | 0.68 |
| Post-menopausal | 21 | 24 | |

(UICC criteria, 1987), ambulatory Karnofsky performance status 80% or above, aged 75 years or less and suitable for primary chemotherapy. The exclusion criteria were: contraindications to combination chemotherapy (cyclophosphamide, vincristine, doxorubicin and prednisolone) and inability to complete quality of life questionnaires. Ninety-seven consecutive women who met these criteria were invited to participate and 96 were willing to give written, informed consent.

All patients underwent bilateral mammography, ultrasound imaging of the breast lesion, fine needle aspiration cytology and/or core biopsy of the breast cancer. Staging investigations carried out were: serum biochemistry, full blood counts and differentials, plain radiographs of chest, pelvis and lumbar spine, isotope bone scans, and abdominal ultrasound (if liver function tests were abnormal).

### Trial design

The basic trial design, including the scheduling of treatment and assessments, is shown in Figure 1. Patients were recruited following the diagnosis and before the start of chemotherapy. They were randomized to standard care (control group) or to standard care plus relaxation training and guided imagery (experimental group). A stratified procedure was used to ensure that there were equal numbers of patients in the two arms of the trial (96 patients were randomized in total). Power calculations showed that a sample size of 96 patients (48 in each group) would have 80% power to detect an effect size of approximately 0.6 (alpha = 0.05, two-tailed).

Following randomization, at 21-day intervals, patients received six cycles of chemotherapy as follows: cyclophosphamide 1 $gm^{-2}$ (maximum dose per cycle: 1.8 g); vincristine 1.5 mg $m^{-2}$ (maximum dose per cycle: 2 mg); doxorubicin 50 mg $m^{-2}$ (maximum dose per cycle: 90 mg), all given as intravenous bolus injections and followed by prednisolone 40 mg orally for 5 days. The dose and timing of chemotherapy was adjusted according to blood white cell count and platelet count.

### Setting

Chemotherapy, relaxation therapy and guided imagery were administered in the Behavioural Oncology Unit, Aberdeen Royal Infirmary, UK. Chemotherapy waiting times were minimized and chemotherapy was normally administered by the same physician. In addition to scheduled appointments for chemotherapy and blood samples, patients were welcome to come to the Unit or to telephone at any time. Staff actively elicited concerns and, if possible, facilitated their resolution. Although we did not arrange formal group meetings, many patients arranged to meet their peers in the Unit to discuss issues of common interest with each other and with the staff. Family members and friends were also made welcome. An informal, but professional, atmosphere was cultivated and we tried to help the women to feel it was ‘their’ Unit.

### Interventions

Patients were taught progressive muscular relaxation and cue-controlled relaxation (Hutchings et al, 1980). Before the first cycle of chemotherapy, patients were issued with audio-cassette recordings containing instructions for relaxation training and they were given a portfolio of ten coloured cartoons to help them visualize their host defences destroying the cancer cells. They were asked to practise at least daily and to keep a daily relaxation diary that contained details of practise frequency, difficulties encountered and a rating of imagery vividness (0–10 scale). The diaries permitted evaluation of the relationship between practise frequency, imagery vividness and response to chemotherapy. In addition, the first 40 women in the experimental group received five live training sessions during the 18 weeks of chemotherapy

### Assessment of clinical and pathological response to chemotherapy

Before each cycle of chemotherapy, and prior to surgery, clinical measurements of the tumour using calibrated skin callipers (four diameters, at 45-degree intervals) (Cheung and Johnston, 1991) were recorded. The measurement before surgery was used in the analysis except in the three patients who died. Response rates were classified, using standard UICC criteria (UICC, 1987). Clinical responses were determined by documenting the product of the two maximal perpendicular diameters (Miller et al, 1981).

Histological responses of excised breast tissue were assessed by one consultant breast histopathologist (IM) as previously documented (Brittenden et al, 1994). Briefly, the response to chemotherapy is graded according to the residual tumour found histologically as follows: type I – changes in tumour cells but tumour nests not destroyed; type II – tumour structure destroyed to a minor degree; type III – tumour structure destroyed to a moderate degree; type IV – tumour structure destroyed to a severe degree; type V – no tumour cells in any of the specimens.

### Psychological assessments

### *Personality and coping*

There is evidence that psychosocial interventions can change how cancer patients cope with stress (Greer et al, 1992; Fawzy et al, 1993). In the present study, coping was assessed using the L-scale of the Eysenck Personality Questionnaire – Revised (EPQ) (Eysenck and Eysenck, 1991) and the Courtauld Emotional Control Scale (CECS) (Watson and Greer, 1983). In a medical context, the L-scale is thought to measure social conformity, high levels of which may represent a coping strategy characteristic of the cancer prone (Type C) personality (Ratcliffe et al, 1995). The

 © Cancer Research Campaign 1999

CECS measures the extent to which individuals cope with dif f emotions (anxiety, anger and unhappiness) by conscious suppression, and this is also thought to be a characteristic of the T personality. To evaluate changes in coping in the two arms, the EPQ-L and the CECS were administered before the first and sixth cycles of chemotherapy.

### *Mood and quality of life*

The primary outcome measures were the Rotterdam Symptom Checklist which yields scores for psychological and physical symptoms (de Haes et al, 1990) and Global Self-rated Quality of Life (GQOL) assessed by means of a five-point Likert scale (ranging from 'very good' to 'very poor').

The Mood Rating Scale (MRS) measures six bipolar dimensions of mood using visual analogue scales with verbally defined anchor points (Table 2). Visual analogue scales have been previously shown to be a reliable method of assessing mood (Bond and Lader, 1974). The dimensions are based on the factor analytic studies underpinning the Profile of Mood States (Lorr and McNair 1984); however, the MRS is much briefer and more acceptable to patients. The MRS is sensitive to changes induced by relaxation and experimental stress in healthy volunteers (Johnson et al, 1996), by chemoimmunotherapy in patients with colorectal cancer (Walker et al, 1997 *a*) and by a weekend 'on-call' in junior medical staff (Wesnes et al, 1997).

The MRS and the RSCL were administered on seven occasions, namely prior to each chemotherapy and three weeks after cycle 6. The GQOL was administered before cycles 1 and 6 only.

### Psychiatric disorder

The Structured Clinical Interview for Diagnosis DSM-III[R] (SCID) (American Psychiatric Association, 1990) was administered by a consultant clinical psychologist or senior trainee in psychiatry to make a psychiatric diagnosis if appropriate at the time of study entry and at the end of chemotherapy. Clinically significant anxiety and depression were assessed using the Hospital Anxiety and Depression Scale (HADS). This scale has been widely used in cancer studies and has been shown to have satisfactory reliability sensitivity and specificity (Zigmond and Snaith, 1983; Razavi et al, 1990; Hopwood et al, 1991; Ibbotson et al, 1994; Hermann, 1997).

### Data analysis and ethics

Data were analysed using SPSS MS for Windows. The conservative 'intention-to-treat' method was used. Patients who died or who were withdrawn from the study, for whatever reasons, were included: their last score for each variable was repeated for all remaining time points. Missing data were minimal. For tests scheduled for seven administrations, the missing data rate was 2.8% and for tests scheduled for two administrations it was 3.1%.

Distributions were screened and transformed if need be to approximate normality. To minimize the risk of a Type 1 error, final scores on the mood and quality of life scales (the two RSCL scales, the six MRS scales and the single-scale GQOL) following chemotherapy were entered into a multivariate analysis of covariance (baseline scores as covariates) (MANCOVA): an identical procedure was carried out for the coping measures (EPQ-L and the three CECS scales). Because both these multivariate analyses were significant, experimental and control groups were then compared using repeated measures analysis of variance (RMANOVA – for variables assessed at two time points) or covariance (RMANCOVA – baseline score as covariate, for variables assessed at seven time points) (Figure 1): Wilks' $\lambda$ was used to evaluate the effect of time and time × group interactions (Tabachnik and Fidell, 1996). The Mantel Haenszel $\chi^2$ test of linear association was used to compare response to chemotherapy in the two groups. Alpha was set at 0.05 (two-tailed).

The study was approved by the Joint Ethical Committee of the Grampian Health Board and the University of Aberdeen.

| Chemotherapy cycle | | 1 | 2 | 3 | 4 | 5 | 6 | |
|---|---|---|---|---|---|---|---|---|
| | | ↓ | ↓ | ↓ | ↓ | ↓ | ↓ | |
| Week | –1 | 0 | 3 | 6 | 9 | 12 | 15 | 18 |
| | EPQ CECS SCID | MRS RSCL HADS GQOL | MRS RSCL HADS | MRS RSCL HADS | MRS RSCL HADS | MRS RSCL HADS | MRS RSCL HADS GQOL EPQ CECS | MRS RSCL HADS SCID |

**Figure 1** Assessment schedule and trial design

**Table 3** Hospital Anxiety and Depression Scores before and after six cycles of chemotherapy

| | | Anxiety | | Depression | |
|---|---|---|---|---|---|
| | | **Before (%)** | **After (%)** | **Before (%)** | **After (%)** |
| Normal | C | 29 (60) | 38 (79) | 46 (96) | 45 (94) |
| (0–7) | E | 31 (65) | 40 (83) | 47 (98) | 43 (90) |
| Borderline | C | 6 (13) | 8 (17) | 2 (4) | 2 (4) |
| (8–10) | E | 10 (21) | 5 (10) | 1 (2) | 5 (10) |
| Abnormal | C | 13 (27) | 2 (4) | 0 (0) | 1 (2) |
| (> 11) | E | 7 (15) | 3 (6) | 0 (0) | 0 (0) |

### RESULTS

### Patients'

The patient details for each group at trial entry are shown in Table 1. The two groups did not differ significantly in terms of age, menopausal status, clinical nodal status and T stage.

There were 74 invasive ductal carcinomas, seven lobular carcinomas and two tubular carcinomas. Ten patients had a complete pathological response and the tumour type, therefore, could not be assessed. Histological assessment of the tumour was not possible in the three patients who died during chemotherapy.

Three patients died, five received fewer than six cycles of chemotherapy, one patient had more than six cycles and one patient withdrew from the study. To determine if these ten patients (deviators) differed from the remaining 86 in terms of baseline variables, the two groups were compared using $\chi^2$ tests and *t*-tests. The two groups did not differ significantly in terms of clinical tumour size at diagnosis ($t$ = 0.59, ns) clinical nodal status ($\chi^2$ = 0.68, ns), ER status ($\chi^2$ = 3.17, ns), psychological treatment (RT and GI versus control) ($\chi^2$ = 0.00, ns), EPQ-L scores ($t$ = 1.89, ns) HADS anxiety ($t$ = 0.67, ns) and HADS depression scores ($t$ = 1.50, ns). Although the ages of the deviators and non-deviators were similar ($t$ = 1.66, ns), a higher proportion of post menopausal women deviated from the protocol ($\chi^2$ = 4.91, $P$ = 0.03).

Forty-six patients received one or more dose reductions. However, dose reduction was not associated with clinical response ($\chi^2$ = 2.34, ns) or with pathological response ($\chi^2$ = 0.29, ns). Similar proportions of patients in the psychological treatment groups ($\chi^2$ = 0.00, ns) received one or more reduced doses of chemotherapy.

The daily diary recordings made each day indicated the following compliance: 23% – less than 0.5 times daily; 29% – 0.5 to 0.99 times daily; 25% – 1 to 1.5 times daily, 23% – more than 1.5 times daily.

### Clinical and pathological responses

The overall clinical response rate after completion of six cycles of chemotherapy was 73% (complete response, 19%; partial response, 54%; stasis, 24%; disease progression, 3%).

After completion of chemotherapy, all patients underwent surgery (except three patients who died). Twenty-three patients had breast conservation and 70 patients had a mastectomy (all had axillary sample or clearance). In 62 of these patients, examination of the excised breast tissue demonstrated the presence of a residual macroscopic lesion. However, this did not necessarily contain tumour cells and in some patients comprised fibrous tissue only. Histological examination of the breast tissue, using the previously defined protocol, demonstrated a type V response (complete histological response with no evidence of residual tumour) in ten patients, type IV in 13 patients, type III in 25 patients, type II in 25 patients and type I in 20 patients.

The two groups did not differ in terms of clinical or pathological response to chemotherapy ($\chi^2$ = 0.94, $P$ = 0.33 and $\chi^2$ = 2.08, $P$ = 0.15, respectively). However, within the experimental group, rating of imagery vividness were positively associated with degree of clinical response (Kendall's $\tau$ = 0.25, $P$ = 0.05).

### Psychological and psychiatric aspects

Psychometric scores at trial entry are shown in Table 2. MANCOVA for the coping measures was significant (Wilks' $\lambda$ $F$ = 3.91, $P$ = 0.006). Means (s.d.) for the two groups before cycles 1 and 6, and the results of repeated measures analyses, are shown in Table 2. The intervention reduced emotional suppression as assessed by the CECS total score (RMANOVA group × time interaction $F$ = 5.24, $P$ = 0.02) and the CECS unhappiness subscale (RMANOVA group × time interaction $F$ = 6.33, $P$ = 0.01) (Table 2). When the control group, the group practising less than daily, and the group practising daily or more were compared, a significant linear relationship was found for CECS unhappiness (RMANOVA group × time interaction $F$ = 3.86, $P$ = 0.03) and CECS total (RMANOVA group × time interaction $F$ = 4.75,$P$ = 0.01); women who practised daily or more had lower scores than control women for CECS unhappiness (RMANCOVA between group $F$ = 4.13,$P$ = 0.01; group × time interaction$F$ = 6.75, $P$ = 0.01) and CECS total (RMANOVA group × time interaction $F$ = 8.64, $P$ = 0.004). EPQ-L scores were not significantly different in the experimental and control groups, although the difference was in the predicted direction (RMANOVAgroup × time interaction $F$ = 2.99, $P$ = 0.09).

MANCOVA for the quality of life measures yielded an overall significant result (Wilks' $\lambda$ $F$ = 2.18, $P$ = 0.03). Means (s.d.) for pre-cycle 1 and 3 weeks, post-cycle 6, and the results of repeated measures analyses, are shown in Table 2 (for GQOL means are for pre-cycles 1 and 6). During chemotherapy, patients receiving the psychological intervention had higher GQOL scores than those in the control group (RMANOVA group × time interaction $F$ = 4.91, $P$ = 0.03): moreover, the GQOL scores of patients in the control group deteriorated significantly during chemotherapy (Student's $t$ = 2.96, $P$ = 0.005). However, this was not the case for those in the experimental group (Student's $t$ = 0.33, $P$ = 0.74). The psychological intervention also enhanced scores of MRS relaxation (RMANOVA between groups $F$ = 3.06, $P$ = 0.01), MRS easy-goingness (between groups $F$ = 5.31, $P$ = 0.02) and MRS total scores (between groups $F$ = 4.82, $P$ = 0.03) (Table 2). RSCL psychological symptom scores were lower in the experimental group (RMANCOVA between groups $F$ = 3.70, $P$ < 0.06) and this reaches significance if EPQ N scores are included as a covariate (RMANCOVA between groups $F$ = 4.04, $P$ < 0.05).

During the study, a number of significant time effects emerged (Table 2). MRS relaxation and MRS confidence increased after the first cycle and then decreased following cycle 6 ($F$ = 3.74, $P$ = 0.004 and $F$ = 2.41, $P$ = 0.04, respectively). MRS energy scores, decreased following cycle 1 and increased following cycle 6 ($F$ = 5.88, $P$ < 0.0005). HADS depression increased steadily until cycle 5 and then declined ($F$ = 4.89, $P$ = 0.001), whereas HADS anxiety declined sharply in both groups prior to the second cycle of chemotherapy and remained at a low level ($F$ = 2.66, $P$ = 0.03) thereafter. RSCL physical symptoms increased markedly following cycle 1 and, thereafter, remained constant until after cycle 6 ($F$ = 11.20, $P$ < 0.0005).

Table 3 shows HADS scores categorized as 'normal' (0–7), 'borderline' (8–10) and 'abnormal' (11 and above) for the two groups during the main phases of the study. The experimental and control groups did not differ significantly before (anxiety $\chi^2$ = 1.00, $P$ = 0.32: depression $\chi^2$ = 0.34, $P$ = 0.56) or after (anxiety $\chi^2$ = 0.04, $P$ = 0.85: depression $\chi^2$ = 0.10, $P$ = 0.75) chemotherapy. There was a strikingly low level of abnormal scores after chemotherapy for anxiety (5%) and depression (0%). Using a cut-off score of 18 for combined scores of anxiety and depression to identify clinically significant disorder (Hopwood et al, 1991), 4% obtained an abnormal score before and 2% after chemotherapy. The two groups did not differ significantly from each other before ($\chi^2$ = 1.04, $P$ = 0.31) or after ($\chi^2$ = 0.00, $P$ = 1.00) chemotherapy. Mean HADS scores (transformed 1 + square root) before and after chemotherapy are shown in Table 2.

At trial entry, only four (4%) patients met the criteria for a SCID diagnosis (two in the control group and two in the experimental group). Two patients had major depression, one patient had panic disorder (without agoraphobia) and one patient (who was on antidepressant medication) qualified for a diagnosis of generalized anxiety disorder and also met the criteria for alcohol abuse.

At the end of chemotherapy, seven (7%) patients received a psychiatric diagnosis (two in the control group and five in the experimental group). Two patients (both in the experimental group) had been given a diagnosis at trial entry. The patient who abused alcohol continued to do so, although she no longer met the criteria for generalized anxiety disorder and she had discontinued antidepressant medication. The second patient who had had major depression now met the criteria for adjustment disorder (depressed type). Of the five new cases, three had major depression (two of whom had a history of major depression) and two had an adjustment disorder (one depressed type and one anxiety type).

### DISCUSSION

Women in both arms of the study showed a high level of enthusiasm and commitment to the project. Compliance with relaxation

 © Cancer Research Campaign 1999

and imagery was good: 77% of the women practised at least once every second day, and 48% at least daily, during the 18 weeks when they were receiving chemotherapy.

Between-group analysis, using the conservative intention-to-treat method, showed that the intervention had beneficial psychological effects: women in the experimental group were more relaxed and easy going (MRS), they had fewer psychological symptoms (RSCL) and they had higher self-rated quality of life (GQOL) during chemotherapy. Because there were so few missing data, an analysis including only those with complete data yielded similar results. This is the first demonstration that relaxation and guided imagery are beneficial for women receiving primary chemotherapy, and the results are consistent with previous studies of relaxation in other groups of cancer patients (Bindemann et al, 1991; Holland et al, 1991; Baider et al, 1994).

The very low level of clinically significant anxiety or depression, whether assessed by means of the SCID or the HADS, in both groups after chemotherapy is striking. The most likely reason for this is the overall milieu provided in the Unit: open access to the Unit, sensitivity to the needs of the patients for information and practical advice, involvement in clinical decision-making to the extent which patients wished, minimization of waiting time for chemotherapy, and the informal, friendly atmosphere (Paraskevaidis et al, 1993; Walker and Eremin, 1996; Fallowfield, 1997). An audit of patient satisfaction at the end of chemotherapy showed that 93% of patients were ‘satisfied’ or ‘very satisfied’ with the emotional support they had received from hospital staff. The results make it clear that a high incidence of clinically significant distress is not inevitable following the diagnosis of breast cancer and during primary chemotherapy. Future studies should address the relative cost-effectiveness of providing a setting such as the above versus a more specialist service that only deals with problems once they have assumed ‘clinical significance.’

Given the low incidence of clinically significant disorder, it is not surprising that the two groups did not differ either in terms of the HADS or the SCID. Moreover, because the high level of satisfaction with emotional support was identical in the two arms (93% of both groups were ‘satisfied’ or ‘very satisfied’ with the emotional support received in hospital), this study provides a rigorous test of the effects of relaxation and guided imagery per se. The effects of the intervention may have been even more striking if the control group had had less support.

As predicted, comparison of the two groups indicated that the intervention had an effect on how patients coped with distress. Following chemotherapy, women in the intervention group were less likely to suppress their emotions, particularly unhappiness (CECS). Moreover, this was most marked in those practising at least daily. The difference between the two groups for social conformity (EPQ-L) did not reach statistical significance at the end of chemotherapy; however, at follow-up 19 weeks later (12 weeks after radiotherapy), women in the experimental group had significantly lower scores than those in the control group ($P < 0.05$). This may be important in the light of our previous finding in patients with Hodgkin’s or non-Hodgkin’s lymphoma that L-scores were a significant independent prognostic factor for 5-year survival (Ratcliffe et al, 1995).

We hypothesized that clinical and pathological responses to chemotherapy would be superior in the experimental group and that the frequency of relaxation practise would be positively correlated with response to chemotherapy. The hypothesis was supported in part. A modest correlation was found between imagery ratings and clinical response. However, there was no evidence that women in the experimental group had an improved clinical or pathological response to chemotherapy.

There were immunological differences between the experimental and control groups. In keeping with the finding of Fawzy et al (1990) that a psychoeducational intervention altered host defences in patients with malignant melanoma, relaxation and guided imagery in this study also had immunological effects, including enhanced lymphokine-activated killer (LAK) cell cytotoxicity, higher numbers (and %) of activated T-cells and reduced blood levels of tumour necrosis factor α (Walker et al, 1997*b*). The intervention, therefore, had a number of biological effects, although the clinical significance of these is not yet clear.

The study design does not permit assessment of the relative contributions of relaxation therapy and guided imagery. Future work is required to clarify this. However, the findings suggest that the combination should be offered to women with breast cancer undergoing primary medical therapy for large and locally advanced breast cancer and this is now routine policy in the Unit.

### REFERENCES

American Psychiatric Association (1990) *Structured Clinical Interview for DSM-III[R]*. American Psychiatric Press Inc: Washington, DC

Baider L, Uziely B and De Nour AK (1994) Progressive muscle relaxation and guided imagery in cancer patients. *Gen Hosp Psychiatry* **16**: 340–347

Bindemann S, Soukop M and Kaye SB (1991) Randomised controlled study of relaxation training. *Eur J Cancer* **27**: 170–174

Bond A and Lader M (1974) The use of analogue scales in rating subjective feelings. *Br J Med Psychol* **47**: 211–218

Bridge LR, Benson P, Pietroni PC and Priest R (1988) Relaxation and imagery in the treatment of breast cancer. *Br Med J* **297**: 1170–1172

Brittenden J, Heys SD, Miller I, Sarkar TK, Hutcheon AW, Needham G, Gilbert F, McKean M, Ah-See AK and Eremin O (1994). Dietary supplementation with L-arginine in patients with breast cancer (>4 cm) receiving multimodality treatment: report of a feasibility study. *Br J Cancer* **69**: 918–921

Burish TG, Vasterling JJ, Carey MP, Matt DA and Krozely MG (1988) Post-treatment use of relaxation training by hypnosis patients. *The Hospice Journal* **4**: 1–8

Cheung CWD and Johnston AE (1991) Carcinoma of the breast: measurement and management of treatment. *Br J Radiol* **64**: 29–36

Coates A, Porzsolt F and Osoba D (1997) Quality of life in oncology practice: prognostic value or EORTC QLQ-C30 scores in patients with advanced malignancy. *Eur J Cancer* **33**: 1025–1030

De Haes JCGM, Van Knippenberg FCE and Niejt JP (1990) Measuring psychological and physical distress in cancer patients: structure and application of the Rotterdam Symptom Check List. *Br J Cancer* **62**: 1034–1038

Downer SM, Cody MM, McCluskey P, Wilson PD, Arnott SJ, Lister TA and Slevin MI (1994) Pursuit and practice of complementary therapies by cancer therapies receiving conventional treatment. *Br Med J* **309**: 86–89

Dreher H (1997) The scientific and moral imperative for broad-based psychosocial interventions for cancer. *Advances: the Journal of Mind-Body Health* **13**: 38–49

Eysenck HJ and Eysenck SBG (1991) *Manual of the Eysenck Personality Scales (EPS Adult)*. Hodder and Stoughton: London

Fallowfield LJ (1997) Offering choice of surgical treatment to women with breast cancer. *Patient Education and Counselling* **30**: 209–214

Fawzy FI, Cousins N, Fawzy NW, Kemeny ME, Elashoff R and Morton D (1990) A structured psychiatric intervention for cancer patients: changes over time and methods of coping and affective disturbance. *Arch Gen Psychiatry* **47**: 720–726

Fawzy FI, Fawzy N, Hyun LS, Elashoff R, Guthrie D, Fahy JL and Morton DL (1993) Malignant melanoma: effects of an early structured psychiatric intervention, coping and affective state on recurrence and survival 6 years later. *Arch Gen Psychiatry* **50**: 681–689

Fraser SCA, Ramirez AJ, Ebbs SR, Fallowfield LJ, Dobbs HJ, Richards MA, Bates T and Baum M (1993) A daily diary for quality of life measurement in advanced breast cancer trials. *Br J Cancer* **67**: 341–346

Greer S, Moorey S, Baruch JD, Watson M, Robertson BM, Mason A, Rowden L, Law MG and Bliss JM (1992) Adjuvant psychological therapy for patients with cancer: a prospective randomised trial. *Br Med J* **304**: 675–680

© Cancer Research Campaign 1999

Gruber BL, Hall NR, Hersch SB and Dubois P (1988) The immune system and psychologic changes in metastatic cancer patients whilst using ritualised relaxation and guided imagery. *Scand J Behav Ther* **17**: 24–46
Gruber BL, Hersh SP, Hall NRS, Waletsky LR, Kunz JF, Carpenter JK, Kverno KS and Weiss SM (1993) Immunological responses of breast cancer patients to behavioural interventions. *Biofeedback Self Regul* **18**: 1–22
Hermann C (1997) International experiences with the Hospital Anxiety and Depression Scale – a review of validation data and clinical results. *J Psychosom Res* **42**: 17–41
Holland JC, Morrow GR, Schmale A, Derogatis L and Stefanek M (1991) A randomised clinical trial of alprazolam versus progressive muscle relaxation in cancer patients with anxiety and depressive symptoms. *J Clin Oncol* **9**: 1004–1011
Hopwood P, Howell A and Maguire P (1991) Screening for psychiatric disorder in patients with advanced breast cancer: validation of two self-report questionnaires. *Br J Cancer* **64**: 353–356
Hutchings DF, Denney DR, Basgall J and Houston BK (1980) Anxiety management and applied relaxation in reducing general anxiety. *Behav Res Ther* **18**: 181–190
Ibbotson T, Maguire P, Selby P, Priestman T and Wallace L (1994) Screening for anxiety and depression in cancer patients: the effects of disease and treatment. *Eur J Cancer* **33A**: 37–40
Johnson VC, Walker LG, Whiting PH, Heys SD and Eremin O (1996) Can relaxation training and hypnotherapy modify the immune response to acute stress, and is hypnotisability relevant? *Contemporary Hypnosis* **13**: 100–108
Lorr M and McNair DM (1984) *Manual of the Profile of Mood States*. Educational and Industrial Testing Service: San Diego
Maguire GP, Lee EG, Bevington DJ, Kuchemann CS, Crabtree RJ and Cornwell CE (1978). Psychiatric problems in the first year after mastectomy. *Br Med J* **1**: 963–965
Miller AB, Hoogstraten B, Staquet M et al (1981) Reporting results of cancer treatments. *Cancer* **47**: 207–214
Paraskevaidis E, Kitchener HC and Walker LG (1993) Doctor–patient communication and subsequent mental health in women with gynaecological cancer. *Psycho-Oncology* **2**: 195–200
Ratcliffe MA, Dawson AA and Walker LG (1995) Eysenck Personality Inventory L-scores in patients with Hodgkin's disease and non-Hodgkin's lymphoma. *Psycho-Oncology* **4**: 39–45
Razavi D, Delvaux N, Farvacques C and Robaye E (1990) Screening for adjustment disorders and major depressive disorders in cancer in-patients. *Br J Psychiatry* **156**: 79–83
Richardson JL, Shelton DR, Krailo M and Levine AM (1990) The effect of compliance with treatment on survival among patients with hematologic malignancies. *J Clin Oncol* **8**: 356–364
Simonton OC, Matthews-Simonton S and Sparks TF (1980) Psychological intervention in the treatment of cancer. *Psychosomatics* **21**: 226–233
Spiegel D, Bloom JR, Kramer HC and Gottheil E (1989) Effects of psychosocial treatment on survival in patients with metastatic breast cancer. *Lancet* **ii**: 888–891
Tabachnik BG and Fidell LS (1996) *Using Multivariate Statistics*, 3rd edn. Harper-Collins: New York
UICC (International Union Against Cancer) (1987) *TNM Classification of Malignant Tumours*. UICC: Berlin
Walker LG (1992) Hypnosis and cancer. *Am J Prev Psychiat Neurol* **3**: 42–49
Walker LG (1998) Hypnosis and cancer: host defences, quality of life and survival. *Contemp Hypnosis* **15**: 34–39
Walker LG and Eremin O (1996) Psychological assessment and intervention: future prospects for women with breast cancer. *Semin Surg Oncol* **12**: 76–83
Walker LG, Walker MB, Heys SD, Lolley J, Wesnes K and Eremin O (1997*a*) The psychological and psychiatric effects rIL-2: a controlled clinical trial. *Psycho-Oncol* **6**: 290–301
Walker LG, Walker MB, Simpson E, Fielden S, Ogston K, Segar A, Heys SD, Ah-See AK, Hutcheon AW and Eremin O (1997*b*) Guided imagery and relaxation therapy can modify host defences in women receiving treatment for locally advanced breast cancer. *Br J Surg* **84**: 31
Walker LG, Heys SD and Eremin O (1998) Surviving cancer: does the fighting spirit help? *J Psychosom Res* (in press)
Watson M and Greer S (1983) Development of a questionnaire measure of emotional control. *J Psychosom Res* **27**: 299–305
Weeks J (1992) Quality of life assessment: performance status upstaged? *J Clin Oncol* **10**: 1827–1829
Wesnes KA, Walker MB, Heys SD et al (1997) What does a weekend 'on call' in a surgical ward do to cognitive performance and mood? *Br J Surg* **84**: 493–495
Zigmond AS and Snaith RT (1983) The Hospital Anxiety and Depression Scale. *Acta Psychiat Scand* **67**: 361–370

 *© Cancer Research Campaign 1999*
